# Preparing the Bone Tissue Regeneration Ground by Exosomes: From Diagnosis to Therapy

**DOI:** 10.3390/molecules25184205

**Published:** 2020-09-14

**Authors:** Batla Al-Sowayan, Farah Alammari, Alaa Alshareeda

**Affiliations:** Stem Cells and Regenerative Medicine Unit, Cell Therapy & Cancer Research Department, King Abdullah International Medical Research Center/King Saud Bin Abdulaziz University for Health Sciences, Ministry of National Guard Health Affairs, Riyadh 11481, Saudi Arabia; alammarifa@ngha.med.sa

**Keywords:** biomaterials, exosomes, bone regeneration

## Abstract

Bone tissue engineering employs acellular scaffolds or scaffolds, along with cells and growth factors, to provide the mechanical support needed, as well as serve as a delivery vehicle for bioactive molecules to the injury sites. As tissue engineering continues to evolve, it has integrated two emerging fields: stem cells and nanotechnology. A paracrine factor that is found to be responsible for the major regenerative effect in stem cell transplantation is an extracellular vesicle called an ‘exosome’. Recent advances in nanotechnology have allowed the ‘exosome’ to be distinguished from other extracellular vesicles and be polymerized into a well-defined concept. Scientists are now investigating exosome uses in clinical applications. For bone-related diseases, exosomes are being explored as biomarkers for different bone pathologies. They are also being explored as a therapeutic agent where progenitor cell-derived exosomes are used to regenerate damaged bone tissue. In addition, exosomes are being tested as immune modulators for bone tissue inflammation, and finally as a delivery vehicle for therapeutic agents. This review discusses recently published literature on the clinical utilization of exosomes in bone-related applications and the correlated advantages. A particular focus will be placed on the potential utilization of regenerative cell-derived exosomes as a natural biomaterial for tissue regeneration.

## 1. Introduction

Large-scale bone tissue defects due to injury, disease, or old age challenge the bone’s natural ability to regenerate itself and require medical intervention. Up till now, an autologous bone grafting approach is considered to be the ‘gold standard’ clinical procedure for bone regeneration [1]. Nevertheless, this approach comes with its drawbacks, as in the ‘best-case scenario’, i.e., no complication, autologous bone grafting will require a secondary procedure for tissue harvesting. This means double the surgical and recovery burden on the patient. Moreover, in some cases, the defected bone area might be large to the extent that it could not be grafted by its own tissue. Therefore, bone tissue regeneration via tissue engineering techniques, utilizing external biomaterials, is being continuously developed and commonly utilized in parallel. There are now different generations of biomaterials including metals, natural/synthetic polymers and ceramics that are used to fabricate scaffolds specifically for bone tissue regeneration [2]. These scaffolds can be simple or composite, i.e., made of more than one type of material. Composite scaffolds, along with additives such as growth factors and stem cells, are now possible to fabricate with the three-dimensional, and the recently introduced four-dimensional bioprinting technology [3]. This has allowed for a more accurate replication of bone tissue biological properties and architecture [4].

Bone healing is a multistep, multifactorial process that involves osteoconduction, osteoinduction and osteogenesis, supported with mechanical stability and revascularization [5]. In general, bone tissue engineering employs scaffolds, along with cells and growth factors, to provide the mechanical support needed, as well as serve as a delivery vehicle for bioactive molecules and drugs. This constitutes the support system needed for the attachment and proliferation of regenerative cells at the injury site [6]. As bone tissue engineering continues to evolve, it has integrated two emerging fields: stem cell and nanotechnology. The stem cell became the star of regenerative medicine, as a large number of studies demonstrated that stem cell transplantation, mostly of mesenchymal stem cells (MSCs), helps improves the functional recovery of different types of damaged tissues, including the bone tissue [7,8,9,10]. Now it is known that these stem cells exert their regenerative effect mainly through its paracrine signaling that induces the targeted tissue to regenerate itself [11,12,13]. It started when one group proposed that the functional improvement in a myocardial infarction animal model, very shortly after bone marrow MSCs transplantation (BMMSCs), could not be attributed to the transplanted cells differentiating into the specialized cells of the damaged myocardium. Instead, it was hypothesized that the transplanted cells exert their tissue regenerative effect by releasing paracrine factors, which stimulate the tissue’s endogenous cells to recover [14,15]. This hypothesis was confirmed when the same group administered the BMMSCs’ condition media alone, and observed similar outcomes as transplanting the whole cells [15]. Then, other groups started reporting the same results, using the same animal model, where stem cell condition media alone was capable of inducing tissue regeneration. Follow up studies on the condition media revealed that the fraction of the media, i.e., the paracrine factor responsible for the major regenerative effect is 100–220 nm in size, and contains ‘exosome’ associated proteins [16,17]. Later on, exosomes were purified from regenerative cell- or stem cell-condition media and tested in animal models with cardiac ischemia. Several studies have consistently reported that exosomes were able to attenuate ischemic tissue damage by inducing endogenous cell repair and promoting angiogenesis [18,19,20,21,22].

Exosomes are nanosized extracellular vesicles [23], that are generated through the endosomal pathway, when the multivesicular endosomes fuse with the plasma membrane and the contained intraluminal vesicles are released into the extracellular space [24]. Exosomes are produced by almost all cell types, under physiological and pathological conditions [25]. This lipid bilayer-enclosed vesicle will carry proteins, lipids, and noncoding RNAs [26], from parent cell to recipient cell. This is done in order for exosomes to initiate biological actions by enabling cell communication, via the message in its cargo, within the cells’ microenvironment or at a distance sight by travelling through the blood circulation. The term exosome was first proposed in 1981, to distinguish this vesicle from other subtypes of extracellular vesicles [27]. Thus, the concept was there decades ago, but there was much confusion around it. Then, in 2014, due to the emerging role of exosomes in biological processes in health and disease, a body called the International Society for Extracellular Vesicles, released a position statement on the minimal experimental requirements for a definition of exosomes [28]. This, along with advances in nanotechnology, have allowed the ‘exosome’ to be polymerized into a well-defined concept. Consequently, in the years after, the published literature that uses the term ‘exosome’ to indicate the extracellular vesicle being examined, usually include an adequate description of the isolation and characterization methodology to justify the nomenclature.

Scientists are now exploring the uses of exosomes in clinical applications. Currently this subtype of extracellular vesicles is being utilized for both diagnostic and treatment purposes. For bone-related diseases, exosomes are being explored as potential biomarkers for different bone pathologies. In addition, they are being exploited as a therapeutic agent where stem cell or progenitor cell-derived exosomes are being used to regenerate damaged bone, either alone or as a part of a composite scaffold. Additionally, progenitor or immune cell-derived exosomes are being tested as immune modulators of bone inflammatory diseases, and finally as drug or biomolecules delivery vehicles. Putting all of this together, this review will discuss the recently published literature on the clinical utilization of exosomes in bone-related pathologies, and the correlated advantages. A particular focus will be placed on the clinical potential of exosomes as up-coming biomaterial for bone tissue engineering.

## 2. Clinical Applications

### 2.1. Diagnosis of Bone-Related Disorders

Exosomes are released into the tissue microenvironment, or blood circulation in order to transport signaling molecules from parent cells to cellular targets. These signaling molecules include proteins, lipids, and noncoding RNAs, and their configuration is highly dependent on the status of the releasing cell and its microenvironment. Therefore, any changes within the microenvironment of a given tissue will be reflected in the content of its released exosomes [29,30,31]. In a number of pathologies, patient blood or body fluid samples were found to contain disease- specific micro RNAs (miRNAs), including bone-disease specific miRNAs [32,33]. For this reason, in addition to the fact that exosome encapsulated miRNAs are highly stable compared to free circulating miRNAs (membrane bilayer protects it from degradation), researchers are now looking into exosomes and their content, including miRNA and other components, as diagnostic and prognostic biomarkers for bone pathologies.

For example, exosomal miRNA was suggested to be used as a biomarker for osteosarcoma. One study reported that serum miR-25-3p levels were significantly upregulated in osteosarcoma patient serum samples, and it correlated with a poor prognosis. In the same study, miR-25-3p was also detected within exosomes secreted by an established osteosarcoma cell line. This indicates that the detected miR-25-3p in patients’ circulation is in tumor-derived exosomes. Therefore, it could be used clinically as a reliable diagnostic and prognostic parameter [34]. Another study investigated the long noncoding RNAs (lncRNA) DANCR expression in serum-derived exosomes as an osteosarcoma biomarker. It reported significantly increased levels of exosomal DANCR in osteosarcoma patient serum samples, compared to samples from patients with benign tumors or samples from healthy donors. The study suggested that this lncRNA DANCR is stabilized by the exosomal membrane, and thus is more reliable as a biomarker than the other tumor-related circulating lncRNAs reported in the literature [35]. Whereas, in osteoporosis, it was reported that plasma exosomal transfer RNA-derived fragments (tRFs)—tRF-25, tRF-38 and tRF-18—could be used as biomarkers for the disease. In addition, it could help discriminate osteoporosis patients with a poor prognosis and high risk of recurrence [36]. Another study reported that osteoporotic patients’ sera contain elevated levels of miR-214-enriched exosomes. The study proposed that these exosomes are released by osteoclasts then up-taken by osteoblasts, which inhibit their function. Therefore, exosome-mediated transfer of miR-214 could be used as a biomarker for bone loss [37]. As for osteoarthritis, it was reported the synovial fluid samples from late-stage patients had a significantly higher expression of exosomal lncRNA PCGEM1 compared to early-stage patients, who in turn had a significantly higher expression of exosomal lncRNA PCGEM1 compared to the control group [38].

On the other hand, a study examining the role of exosomes in bone aging, reported that the aged osteocytes, compared to the young controls, release exosomes that contain elevated levels of proinflammatory proteins. These findings were based on a proteomics analysis of exosomal protein contents. These elevated proteins play a role in regulating innate and adaptive immunity, wound healing, and angiogenesis in addition to eliminating oxidative stress [39]. This indicates that aged osteocytes-released exosomes will act as shuttles to transfer factors that promote a proinflammatory state, a key characteristic of bone aging. Thus, exosomal protein composition could not only be used to examine and measure bone aging, but also to analyze the exosomal content, proinflammatory vs. anti-inflammatory, and can be used as a ‘bone wellness’ assessment tool for personalized medicine and treatment monitoring.

### 2.2. Treatment of Bone-Related Disorders

The bone tissue undergoes continuous maintenance and renewal processes. The role of exosomes in the biological actions related to bone remodeling is being investigated. It is believed that osteoblasts, osteocytes, and osteoclasts, in addition to the other cell types involved, communicate in part through the release of exosomes [40]. This cellular communication enables the intricate, multistep processes of bone remodeling to be fulfilled. As the role of exosomes within the bones’ microenvironment becomes better understood, scientists are experimenting with the possible therapeutic applications of these ‘shuttle vesicles’. In general, and for bone-related disorders, exosomes are employed in three main therapeutic purposes; they are either used to initiate tissue repair, or to modulate immune reaction, or to be used as a vehicle for therapeutic agents. For each application, the exosome source, i.e., parent cell, must be carefully selected in order for this vesicle to perform its intended action. More details regarding these applications in relation to bone pathologies are discussed in the following sections.

#### 2.2.1. Bone Tissue Regeneration

In regenerative medicine, scientists have utilized stem cell transplantation, mainly MSCs, to regenerate damaged tissues including bones [41]. However, there is a shift now from the ‘whole cell’ based approach into a more customized tactic. The new approach encompasses the injection of the active cellular components that initiate tissue regeneration, rather than the entire cell. A growing body of literature employs the injection of regenerative cell-derived exosomes to elucidate tissue regeneration [42]. This shift from ‘whole cells’ to ‘exosomes’ is due to the fact that the exosome, with its action inducing cargo, is believed to be the key player in tissue regeneration. Cell-fee exosomes were shown, in a number of studies, to initiate the repair of different body tissues including skin [43,44], cartilage [45], pancreatic [46], and cardiovascular tissues [47]. In addition, there are more than a few advantages of exosome injection versus cell transplantation. These include the fact that these extracellular vesicles are nonviable, which means that they will not replicate. Unpredictable replication, differentiation, and possible DNA mutations are a risk in cellular therapy that could lead to tumor formation [48]. Second, being acellular, an exosome derived from a regenerative cell is less likely to trigger an immune reaction and rejection compared with its parent cell [49]. This will allow for the recurrent administration of therapeutic exosomes, i.e., as required to achieve the therapeutic goal, without needing to administer immunosuppressive drugs in parallel. Third, exosomes are released with membrane-bound proteins that can be easily engineered to target a given tissue, including bone, and be incorporated into its cells [50,51]. Poor engraftment within the targeted tissue and accumulation of transplanted cells in nontargeted organs, mostly the lungs, are also issues in cell-based therapies [52,53]. Fourth, since exosomes are nanosized, they can navigate easier through tissue barriers, including the blood–brain barrier [54], which is very crucial when the target is within the central nervous system. Fifth, physical elements of the tissue microenvironment, such as low oxygen tension in an ischemic tissue, affect the cell and the nature of its released paracrine factors including exosomes. However, directly injecting exosomes into an ischemic tissue will likely have no effect on this vesicle and its cargo. Therefore, exosomes could be a better option when there is a critical-sized tissue defect, as transplanted cells in such cases could be affected by the microenvironment [55,56]. Due to all these advantages, scientists studying bone tissue regeneration are starting to utilize exosomes in a number of bone-degenerative diseases. The examined exosomes are of different cellular origins and are injected alone or administered as a part of a scaffold.

There are several in vitro studies investigating the potential use of exosomes as a bone-regenerating tool. These studies have repeatedly shown that exosomes, of different cellular origins, have the capacity to induce osteogenic differentiation via a number of proposed mechanisms. One study preconditioned adipose tissue-derived stem cells (ASCs) with tumor necrosis factor-alpha (TNF-α). This was done to mimic the acute inflammatory phase during bone injury. The study reported that exosomes from both preconditioned and nonconditioned cells significantly increased osteoblast differentiation. However, exosomes derived from TNF-α-preconditioned ASCs had a stronger effect. This was proposed to be, at least in part, due to the fact that these exosomes had a higher content of Wnt-3a, a crucial modulator of bone tissue metabolism [57]. A study on exosomes derived from MSCs, at different time points of osteogenic differentiation, reported that these exosomes promoted MSCs osteogenic differentiation in a stage-dependent manner. Then, miRNA profiling revealed that exosomes derived from cells at an early-stage of osteogenic differentiation had significantly different miRNA contents compared to exosomes derived from cells at a late-stage of differentiation [58]. Another study incubated MSC-derived exosomes with BMMSCs derived from rats with steroid-induced femoral head necrosis (SFHN). Exosomes derived from the healthy BMMSCs suppressed the adipogenic commitment by the necrotic BMMSCs, which in turn increased osteogenesis. The study proposed that this is due to upregulated Sox9 protein levels within the exosomes, as Sox9 silencing significantly reduced the exosomes’ osteogenic effect [59]. Whereas, a study reported that myoblast-derived exosomes increased the osteogenic differentiation of preosteoblasts. The proposed mechanism of action was that the muscle miR-27a-3p content of myoblast-derived exosomes decreased the adenomatous polyposis coli (APC) expression in recipient cells, which activated the β-catenin pathway [60]. Another study suggested that preosteoblast-derived exosomes induced osteogenic differentiation of the same cells through their miR-let-7 content. This was confirmed when exosomes treated with a let-7 inhibitor had a reduced effect on the cells [61].

In addition, in vitro studies have also reported that stem cell-derived exosomes have the capacity to induce osteoblast/osteocytes proliferation and protect against apoptosis through, again, different proposed pathways. When ASCs were treated with low-level laser irradiation, their released exosomes had an increased expression of the antiapoptotic protein Bcl-2, and a decreased expression of the proapoptotic protein Bax. As a result, when these exosomes were added to osteocytes cultured under hypoxia, cell apoptosis was significantly inhibited [62]. The study on the TNF-α-preconditioned ASCs reported that the elevated Wnt-3a exosomal content, in addition to increasing differentiation, increased osteoblasts proliferation [57]. Whereas, studies on BMMSC-derived exosomes reported that the treated osteoblasts had an increased proliferation and decreased apoptosis due to these exosomes expressing key proteins of the bone-forming MAPK signaling pathway, including p-p38 and p-JNK [63,64]. Validating these reports, a recent in vivo study employing a unilateral tibial distraction osteogenesis animal model on old rats reported similar outcomes. The study showed that when exosomes isolated from the BMMSCs of younger rats were injected into the distraction gaps, bone regeneration was markedly accelerated. This was based on digital radiography, microcomputed tomography, histological analysis and mechanical testing. However, the mechanism of young BMMSC-derived exosomes’ action on the old BMMSCs’ proliferation and osteogenic differentiation was unclear [65].

Other in vivo studies on bone regeneration have utilized cell-derived exosomes combined with other biomaterials. For example, one study used exosomes derived from induced pluripotent stem cell-derived MSCs, in combination with tricalcium phosphate (β-TCP). First, an in vitro investigation revealed that MSC-derived exosomes increased BMMSCs proliferation, migration, and osteogenic differentiation. Then, a classical porous β-TCP scaffold was used as an exosome carrier and implanted into a critical-sized calvarial defect animal model. A histological and immunohistochemical analysis revealed that MSC-exosome-β-TCP scaffolds implantation resulted in greater new bone formation compared to pure β-TCP scaffolds. Based on gene expression profiling and bioinformatics analyses, the study proposed that MSC-exosomes induce β-TCP osteoinductivity by activating the PI3K/AKT signaling pathway [66]. Another study combined ASC-derived exosomes with poly (lactic-co-glycolic acid) (PLGA) scaffolds. Initial in vitro testing showed that ASC-exosomes increased BMMSC proliferation, migration and osteogenic differentiation. Then, in vivo testing utilizing a critical-sized calvarial defect animal model, followed by microcomputed tomography and histological assessments, showed that the ASC-exosome-PLGA scaffold significantly enhanced bone regeneration compared to exosome free PLGA. Immunofluorescence staining of scaffolds, a week after implantation, revealed that ASC-exosome-PLGA recruited around 2-fold more MSCs, compared to exosome free PLGA [67]. A third, in vitro only, study combined MSCs, their exosomes, and a titanium disc. Exosomes were purified from the condition media of MSCs that were cultured under standard cell culture conditions or osteogenic differentiation conditions. Then, these exosomes were mobilized on the titanium discs and MSCs were added. Immobilization of exosomes, from both cell groups, on the titanium disc increased MSCs adhesion, spreading and proliferation. Based on a quantitative polymerase chain reaction analysis, MSCs cultured on the exosome–titanium disc had an upregulated stromal cell-derived factor (SDF-1α) expression. SDF-1α is a cell recruiting factor that could, in vivo, induce endogenous MSCs migration towards implantation site, thus boosting tissue regeneration [68] (see Summary in Table 1).

#### 2.2.2. Immune Modulation

As in tissue regeneration, it is believed that MSCs regulate inflammatory responses through the release of paracrine factors, with the most crucial being exosomes [69,70,71]. Therefore, immune modulation studies are employing an exosome-based, cell-free-approach to develop novel therapeutics for a number of inflammation-based pathologies. The use of an extracellular vesicle versus the use of an entire cell to regulate the immune system could be advantageous due to the previously mentioned reasons. One of the most common bone-related inflammations is osteoarthritis (OA). In which, inflammation within the synovial capsule causes a loss of articular cartilage which in turn leads to joint degeneration, and sclerotic changes to the surrounding bone tissue [72]. Studies have shown that MSC-exosomes induce healing by promoting chondrocytes proliferation and migration, which leads to cartilage regeneration and the protection of the underling bone tissue [73]. Another reported exosomal action that is crucial in OA management is restoring the synovial homeostasis, which is essential for tissue healing and repair. For example, one group reported that the therapeutic effect of MSC-exosomes in an OA animal model is a result of exosomes enhancing synthesis and suppressing the degradation of the chondrocyte extracellular matrix (ECM). This was confirmed via an in vitro investigation on chondrocytes treated with the proinflammatory cytokine interleukin (IL)-1β, where exosomes increased collagen type II synthesis and decreased the expression of ADAMTS5, an ECM degrading enzyme [74]. A second study reported that MSC-exosomes protected OA mice from joint damage by inducing chondroprotective actions. An in vitro model of chondrocyte culture revealed that MSC-exosomes induce the cellular expression of type II collagen and aggrecan, and at the same time dissuade the cellular expression of catabolic markers MMP-13 and ADAMTS5, and the inflammatory marker iNOS [75]. A third study proposed that the MSC-exosome-mediated OA joint repair in rats is a result of adenosine activation of the signaling pathways—AKT, ERK and AMPK. This was confirmed when the chondroprotective effects were revoked after using inhibitors of adenosine receptor activation, and AKT/ERK/AMPK phosphorylation [76]. A fourth in vivo study on an osteochondral defect animal model reported a similar mechanism of action, where the repair was mediated by MSC-exosomes, particularly their CD73 content, activating AKT and ERK signaling pathways. Again, inhibitors of AKT/ERK phosphorylation attenuated the exosome-mediated cell proliferation and migration in vitro. The study also reported that MSC-exosomes promoted tissue repair in defected areas by promoting higher infiltration of tissue repairing CD163^+^ M2 macrophages over inflammatory CD86^+^ M1 macrophages, in addition to reducing proinflammatory cytokines IL-1β and TNF-α [77].

Research groups have also investigated the immunomodulatory effect of MSC-exosomes in association with other biomaterials and reported similar outcomes. A study used cartilage ECM/gelatin methacrylate (GelMA)/BMMSC-exosome scaffolds in an osteochondral defect animal model. They reported that the exosome-coated scaffolds induced cartilage regeneration by a number of actions including polarizing synovial macrophages towards an M2 phenotype. In addition, it was also reported that BMMSC-exosomes protected chondrocytes from oxidative stress in the degenerative tissue by restoring mitochondrial dysfunction through the supplementation of mitochondrial-related proteins [78]. Another study used polycaprolactone (PCL)/Poly(dopamine) (PDA)/*S*-nitrosoglutathione (GSNO)/BMMSCs-coated scaffolds. They reported that this composite scaffold not only induced the osteogenic differentiation of BMMSCs, but also had an immunoregulatory action. This was concluded when lipopolysaccharide-treated macrophages were cocultured with the exosome-coated scaffold, and as a result, they were significantly elongated and their expression of proinflammatory genes (IL-1β, iNOS, TNF-α, and IL-6) was markedly reduced [79]. The last study took a different approach, where they isolated exosomes from macrophages that were pretreated with bone morphogenetic protein 2 (BMP2). The BMP2-macrophages-exosomes were used to coat titanium nanotube implants. The coated implants were incubated with BMMSCs in a coculture system. The results revealed that exosomes increased the biofunctionality of titanium nanotubes, as BMMSCs incubated with exosome-coated implants had an increased expression of the osteoblast differentiation markers, alkaline phosphatase and BMP2, compared to BMMSCs cocultured with exosome-free implants. In addition, exosome/titanium implants induced cytokine profile changes within BMMSCs, these cytokines included fibroblast growth factor-19, IL-17A, growth/differentiation factor-15, IL19, interferon gamma-induced protein (IP)-10, macrophage inflammatory protein (MIP)-3a, and RANTES [80] (see Summary in Table 2).

#### 2.2.3. Drug Delivery

As cargo carriers and transporters by nature, exosomes are being employed for the targeted delivery of drugs and biomolecules. Nanoparticles are used as delivery vehicles in order to improve the efficiency of therapeutic agents. Exosomes are now being explored for this purpose due to their possible advantages. Compared to other synthetic nanoparticles, exosomes possess few favorable traits including the fact that these extracellular vesicles are designed by nature to mediate short or long-distance cellular communication. This is done by carrying cargo, proteins or noncoding RNAs, from the releasing cell to the recipient cell, while maintaining its stability when travelling through the body. Therefore, the cup-shape structure and the bilayer membrane is designed to be loaded with different therapeutic agents. Second, as mentioned earlier, exosomes can negotiate efficiently across different tissue barriers, allowing delivery to different tissue targets. Third, exosomes are natural biological structures therefore they are not cytotoxic and well tolerated by the immune system. Exosomes, especially those collected from own cells, are less likely to trigger immune responses compared with other materials, thus loaded drugs or biomolecules are protected from elimination by the immune cells [81]. Fourth, as previously discussed, exosomes have an intrinsic tissue targeting ability. They are released with membrane lipids and proteins that will direct exosomes to a specific tissue target. Furthermore, exosomes could be specifically engineered for their target, by manipulating the parent cell or the exosome itself to increase the targeting capacity [82,83]. Then, when an exosome reaches its cellular target, its membrane naturally fuses with the cell’s membrane—this mode of entry of exosomal cargo into the cell—help preserves the integrity of the delivered drug or biomolecule [84].

In drug delivery studies, exosomes are mostly employed in cancer therapies. Exosomes derived from different cell types are loaded with anticancer drugs or noncoding RNA for the purpose of cancer management and prevention of metastasis. Anticancer drugs are loaded into exosomes in an attempt to improve tumor targeting, increase drug stability, reduce the cytotoxicity of the drug to other body tissues, and reduce a build-up of resistance towards that drug [85,86,87,88]. For bone-related cancers, one study loaded BMMSC-derived exosomes with the chemotherapeutic drug doxorubicin and examined its effect on an osteosarcoma cell line. An in vitro investigation revealed that cells up-take exosome-loaded-doxorubicin more efficiently compared to free-doxorubicin. This led to an increase in cell death and an overall better anticancer effect. Simultaneously, exosome-loaded-doxorubicin was less toxic to noncancerous cells as revealed by in vitro testing on a myocardial cell line [89]. Another study investigated the effect of miR-101 on osteosarcoma invasiveness and metastasis. In order to deliver the therapeutic molecule to its target, the research group transduced ASCs with lentiviral particles to overexpress miR-101, and thus release miR-101-enriched-exosomes. First, in vitro testing showed that miR-101, delivered via exosomes, reduced the invasion and migration of the tested osteosarcoma cell line. Then, the lung metastasis animal model of osteosarcoma showed that the systemic administration of the miR-101-enriched-exosomes suppressed lung metastasis with no apparent side effects [90].

## 3. Closing Remarks

Bone tissue regeneration using external biomaterials as acellular scaffolds or cell-seeded scaffolds were shown to be effective. These scaffolds provide the damaged bone tissue with the mechanical support needed for it to regenerate itself, and in addition serve as a platform to deliver therapeutic agents to the site of implantation. Scaffold fabrication techniques continue to evolve as new methodologies and biomaterials are introduced in a constant effort to improve their effectiveness. On the other hand, recent studies in the field of bone tissue regeneration have established that exosomes are key mediators within the bone microenvironment. It was consistently shown that the progenitor or immune cell-derived exosomes are able to induce both bone-regenerative and bone-protective effects. A number of mechanisms of action were proposed, but the exact mechanism of the exosome-mediated signaling cascade for bone remodeling is yet to be fully understood.

Many reported that the injection of exosomes alone, or the addition of these exosomes into scaffolds implanted in bone-defect animal models, improved the bone tissue regeneration capacity. This was a result of exosomes inducing the proliferation and osteogenic differentiation of endogenous progenitor cells. In addition, it is believed that the increased regenerative capacity was also a result of these exosomes improving the immune tolerance of the implanted scaffolds (Table 1). Whereas, in animal models of bone-related inflammations, it was reported that exosomes helped restore the homeostasis of inflamed bone microenvironments by inducing cell-protective and anti-inflammatory actions on neighboring chondrocytes and macrophages (Table 2). This combined action of regenerative/immune modulatory actions of exosomes, mostly MSC-derived exomes, will be rendered very valuable for the purpose of tissue regeneration, as it is well established by now that restoring the normal physiology of the tissue microenvironment is the first step in tissue healing.

Another clinical application for these extracellular vesicles is to utilize them as carriers for other therapeutic agents. Exosomes are carriers by nature and are well equipped to travel across different tissue barriers. Researchers successfully loaded exosomes with synthetic drugs, mostly anticancer drugs. Additionally, studies have been successful in engineering parent cells to overexpress a specific biomolecule, i.e., miRNA, so that the released exosomes will become enriched in this particular therapeutic biomolecule. Both approaches, as discussed earlier, were successful in bone-related therapies. The third clinical application for exosomes is to be used as a biomarker for disease diagnosis and prognosis. This is due to the fact that disease-specific miRNAs that are encapsulated within exosomes are more stable compared to free circulating miRNAs. Therefore, the content of exosomes isolated from blood samples, or other body fluid samples, could be used as reliable biomarkers for bone disease, bone aging and general bone wellbeing as demonstrated by the cited literature.

Recent developments in exosome engineering indicate the potential of producing cell-free, nanoscale particles with customized surface proteins and cargo to target a specific tissue and elucidate a desired action. Considering the reported benefits of exosomes compared with other cell-based and noncell-based tissue regeneration methods, exosome-based therapies could be of significant interest in promoting effective bone tissue regeneration and prevent immune-related bone damage. Exosomes can be mass-produced and packaged as an off-the-shelf product such as any biomaterial used in scaffold fabrication. This will make it very feasible to fabricate scaffolds with immobilized exosomes integrated into them. Exosomes’ parent cells and concentration could be chosen depending on the patient needs and intended purpose, e.g., progenitor cell-derived exosomes to promote cell proliferation or immune cell-derived exosomes to prevent an immune reaction. In addition, exosome-containing injections could be given following scaffold implantation to improve tissue healing in severe cases. The injected exosomes, again dependent on patient needs, could be natural cell-derived exosomes, exosomes derived from engineered cells with specific miRNA content, or drug-loaded exosomes.

All in all, the literature on the use of exosomes for bone tissue regeneration either as a key player in the regeneration and recovery process or as a carrier of therapeutic molecules, or as a biomarker, all report consistently promising outcomes for this extracellular vesicle. Nevertheless, for this relatively new application to realize its full potential further investigations are expected to address a number of issues. These include (1) the triggers and mechanisms that regulate the assembly of specific bioactive molecules inside these vesicles, (2) the signals that activate their release, (3) the surface receptors that confer their tissue target specificity, (4) the mode of internalization by recipient cells of targeted tissue, and (5) finalization of quality control issues regarding the technical aspects of exosome isolation, storage, and mode of administration.

## Figures and Tables

**Table 1:** Summary of published work on exosomes utilization for bone tissue regeneration.

Exosome Source	Testing Model	Action	Proposed Mechanism of Action	Ref.
Human ASCs, preconditioned with TNF-α	In vitro; Human osteoblastic cells	Increased cell proliferation and osteogenic differentiation	The effect was attributed to Wnt-3a exosomal content that was elevated when cells were preconditioned by TNF-α.	[57]
Human MSCs cell line, at different time points of osteogenic differentiation	In vitro; Human MSCs cell line	Induced osteogenic differentiation in a stage-dependent manner	Exosomes from different stages of differentiation have different miRNA content (up to 16 significantly altered miRNAs); these miRNAs are involved in pathways related to osteogenic differentiation.	[58]
Rat BMMSCs	In vitro; BMMSCs from rat with SFHN	Increased osteogenic differentiation	The effect was attributed to high Sox9 exosomal content; this was confirmed when Sox9 silencing significantly decreased the osteogenic effect.	[59]
Mouse myoblasts cell line	In vitro; Murine preosteoblasts cell line	Increased osteogenic differentiation	Exosomes had high levels of miR-27a-3p, which when up taken by recipient cells, decreased APC expression, thus activating β-catenin pathway.	[60]
Murine preosteoblasts cell line	In vitro; Murine preosteoblasts cell line	Increased osteogenic differentiation	The effect was attributed to the miR-let-7 exosomal content; this was confirmed when the addition of a let-7 inhibitor resulted in a reduction in osteogenesis.	[61]
Mouse ASCs, treated with low-level laser irradiation	In vitro; Murine osteocytes-like cell line	Apoptosis was inhibited when exosomes were added to cells cultured under hypoxia	Exosomes increased cellular expression of anti-apoptotic protein, Bcl-2, and decrease expression of pro-apoptotic protein, Bax.	[62]
Rat BMMSCs	In vitro; Human fetal osteoblastic cell line	Increased cell viability and proliferation	Cellular expression levels of key proteins in the MAPK pathway, p-p38 and p-JNK, were significantly upregulated.	[63,64]
Young rat BMMSCs (2 weeks)	In vitro; Old rat BMMSCs (15 months) Animal model; Unilateral tibial distraction osteogenesis on old rats	Increased proliferation and osteogenic differentiation Exosomes injection into the distraction gaps markedly accelerated bone tissue regeneration	Young BMMSC-derived exosomes promote bone regeneration through enhancing the proliferation and osteogenic capacity of BMMSCs.	[65]
Human MSCs	In vitro; Human BMMSCs Animal model; Critical-sized calvarial bone defect on rats, Exosomes blotted onto β-TCP scaffolds	Increased proliferation, migration and osteogenic differentiation β-TCP/exosome implanted into the defect areas had better osteogenesis action than β-TCP scaffolds	Exosomes enhance the osteoinductivity of β-TCP through activating the PI3K/AKT signaling pathway of BMMSCs.	[66]
Human ASCs	In vitro; Human BMMSCs Animal model; Critical-sized calvarial bone defect on mice, Exosomes blotted onto PLGA scaffolds	Increased proliferation, migration and osteogenic differentiation PLGA/exosomes resulted in more bone tissue formation compared to exosome free scaffolds	Exosomes enhance the osteoinductivity of PLGA scaffolds by promoting BMMSCs migration and engraftment onto the newly formed bone tissue.	[67]
Human ASCs	In vitro; Human ASCs, Exosomes immobilized on titanium discs	Exosomes immobilization promoted cell adhesion, spreading and proliferation on the titanium discs	Exosomes were enriched in cell adhesion and signaling molecules. Additionally, they upregulated SDF-1α gene expression, a cell recruitment factor, in cells cultured on the titanium surface.	[68]

**Table 2:** The immune modulatory effects of mesenchymal stem cell (MSC)-exosomes in relation to cartilage regeneration and bone protection.

Cells Affected	Immunomodulatory-Tissue Regenerative Action	Ref.
**Chondrocytes**	MSC-exosomes restored microenvironment homeostasis by inducing cell protective and anti-inflammatory actions on chondrocytes, including: Activation of regulator pathways (e.g., AKT, ERK and AMPK) Increasing chondrocyte markers expression (e.g., type II collagen and aggrecan) Inhibiting catabolic markers expression (e.g., MMP-13 and ADAMTS5) Inhibiting inflammatory markers expression (e.g., iNOS) Promoting mitochondrial recovery, by supplementing mitochondrial-related proteins.	[74,75,76,77,78]
**Macrophages**	MSC-exosomes modulate synovial macrophages towards regenerative, anti-inflammatory phenotype by: polarization of macrophages towards M2 phenotype over M1 phenotype Reducing proinflammatory cytokines gene expression and release (e.g., IL-1β, TNF-α, iNOS and IL-6).	[75,77,78,79]

## Abbreviations

MSC	Mesenchymal stem cell
BMMSC	Bone marrow MSC
miRNA	Micro RNA
lncRNA	Long noncoding RNA
tRFs	transfer RNA-derived fragments
ASC	Adipose tissue-derived stem cell
TNF-α	Tumor necrosis factor
SFHN	Steroid-induced femoral head necrosis
APC	Adenomatous polyposis coli
β-TCP	Tricalcium phosphate
PLGA	poly (lactic-co-glycolic acid)
SDF-1α	Stromal cell-derived factor
OA	Osteoarthritis
ECM	Extracellular matrix
IL	Interleukin
BMP2	Bone morphogenetic protein 2
